# Assessing the Potential for a Malaria Outbreak in Trinidad and Tobago Due to Migrant Populations From Guyana and Venezuela for the Period 2009–2019

**DOI:** 10.7759/cureus.96130

**Published:** 2025-11-05

**Authors:** Reshma Narinesingh-Motie, Marsha Ivey, Jonathan Edwards, Robert Jeffrey Edwards

**Affiliations:** 1 Insect Vector Division, Ministry of Health Trinidad and Tobago, Cunupia, TTO; 2 Faculty of Medical Sciences, University of the West Indies, Trinidad, TTO; 3 Research Department, Medical Research Foundation of Trinidad and Tobago, Port of Spain, TTO

**Keywords:** guyana, malaria, migrants, outbreak, trinidad and tobago, venezuela

## Abstract

Objective: This study aims to assess the risk of a malaria outbreak in Trinidad and Tobago (T&T) based on trends in imported cases from two malaria-endemic high-population-mobility countries, Guyana and Venezuela, for the period 2009-2019.

Methods: A census of all positive malaria cases in T&T for the period 2009-2019 was obtained from the Insect Vector Control Division of the Ministry of Health. This included sociodemographic data, malaria type, origin, and whether it was acquired locally or was imported. Over the study period, data on the number of arrivals from Guyana and Venezuela to all ports of entry were obtained from the Immigration Division. Descriptive analyses and linear regression analyses were used to investigate the relationship between positive malaria cases and Guyanese and Venezuelan arrivals.

Results: There were 211 positive malaria cases in the study; ages ranged from 3 to 81 years, with a mean age of 35 years (SD = 14.3), and 74% were males. Among malaria cases, the common parasites were *Plasmodium vivax* (68.2%) and *Plasmodium falciparum* (22.3%). The majority (91.9%) were imported, 6.2% were acquired locally, and 1.9% were of unknown origin. Of the 194 imported cases, the countries of origin were Guyana (39.7%), Venezuela (38.7%), and Sub-Saharan Africa (15.5%). The correlation between log-transformed Venezuelan arrivals and malaria cases was moderate (r = 0.642, R^2^ = 0.412) and reached statistical significance (p = 0.033), indicating that Venezuelan arrivals significantly predict malaria incidence.

Conclusion: Population movement from high malaria risk areas may increase the likelihood of transmission in T&T, and the influx of people from Venezuela significantly predicts malaria incidence. Malaria literacy, health education, surveillance, prompt diagnosis, treatment, and vector control are some of the interventions urgently needed to prevent a malaria resurgence.

## Introduction

In 2023, malaria caused approximately 263 million infections and 597,000 deaths worldwide [1]. Malaria is a life-threatening, vector-borne disease caused by the protozoan parasite, *Plasmodium* species, transmitted to humans by the bite of a female *Anopheles* mosquito [1]. Infections are preventable and treatable and occur predominantly in the tropical and subtropical regions of the world. Five *Plasmodium* species affect humans: *Plasmodium falciparum*, *Plasmodium vivax*, *Plasmodium ovale*, *Plasmodium malariae*, and *Plasmodium knowlesi* [2]. Of these, *P. falciparum* and *P. vivax* are the greatest threats to human life. *P. falciparum* is the dominant malaria parasite in Africa, and *P. vivax* is the most common in Latin America and the Caribbean region, accounting for 74% of the infections [1]. *P. vivax* is the most difficult form to treat and manage, as the parasite places dormant hypnozoite forms in the liver that may result in multiple relapses, thus presenting many challenges to treatment regimens and control [3]. The main methods recommended by the World Health Organization (WHO) for malaria prevention include vector control, chemoprevention, and personal protective measures to prevent mosquito bites [1].

Trinidad and Tobago (T&T) was certified malaria-free by the WHO in 1965, meaning there was the interruption of local transmission of malaria cases with zero indigenous cases for at least three consecutive years [1]. T&T is not endemic for malaria; thus, the number of confirmed cases every year is imported and/or introduced [4]. An imported case of malaria infection is one in which the infection was acquired outside the area where it was clinically manifested, diagnosed, and managed [5], as determined by investigation of the patient's travel history to endemic malaria areas through epidemiological surveillance. The Insect Vector Control Division (IVCD) of the Ministry of Health is the organization that oversees testing, treatment, and follow-up of patients, integrated vector management, and serves as the reference laboratory for malaria diagnosis within T&T [4]. Surveillance using morphological techniques has confirmed *Anopheles *mosquito populations throughout Trinidad that are competent vectors for malaria transmission. *Anopheles aquasalis* was associated with a cluster of introduced *P. vivax* malaria cases in T&T in 1990-1991 [6], and in 1994-1995, an autochthonous focus of *P. malariae* cases was found to be associated with *Anopheles homunculus* and *Anopheles bellator* [6].

T&T is considered an upper-middle-income developing country that attracts tourists and migrants seeking better economic opportunities and improved living conditions [7,8]. Venezuela, the closest neighbor to T&T, is a malaria-endemic country [9] that has been experiencing political and economic instability since 2014 [8]. The short distance of 7 miles between the eastern coast of Venezuela and the southern coast of Trinidad makes it a more easily accessible location by both sea and air. Beginning in 2017, T&T has been inundated by the Venezuelan displacement crisis, and as of 2022, it was estimated that there were approximately 34,100 migrants, refugees, and asylum seekers in the country [7]. T&T, with a population of approximately 1.36 million people in 2023 [10,11], has received more Venezuelans per capita than most other host countries in the region [8]. Guyana is a malaria-endemic country [12] that shares a border with Venezuela and is approximately 432 miles from Trinidad's southern coast, with travel to T&T primarily via air. Although not experiencing political upheaval, nationals of Guyana may work in T&T for economic reasons due to their membership in the CARICOM Single Market and Economy (CSME), which facilitates the uninhibited movement of skilled personnel, capital, and businesses among participating Caribbean member states [13].

In the Caribbean region, among the CSME member states, Guyana remains the only malaria-endemic country, as Belize and Suriname were certified malaria-free in 2023 and 2025, respectively [14]. The free movement of people facilitated by the CSME allows nationals from malaria-endemic countries to enter T&T [12,13]. Studies have shown that the resurgence of malaria in Venezuela has acted as an epidemiological reservoir, fueling outbreaks in Brazil, Colombia, and Peru through migration, mining activities, and weakened surveillance [15-17]. Regional population mobility increases the potential risk of malaria reintroduction in T&T. The aim of the study was to assess the risk of a malaria outbreak in T&T by examining trends in imported cases from two endemic countries, Guyana and Venezuela, for the period 2009-2019.

## Materials and methods

Study design and setting

This retrospective observational study investigated malaria cases and port arrivals in T&T for the period 2009-2019. Data for malaria cases were obtained from the IVCD of the Ministry of Health. T&T port arrivals were obtained from the Immigration Division of the Ministry of National Security.

The study was approved by the Campus Research Ethics Committee of the University of the West Indies, St. Augustine, with approval number CEC1205/07/19, in accordance with the revised Helsinki Declaration 2000.

Sampling strategy

The study included all positive malaria cases in T&T from 2009 to 2019, including those who were "in-transit." A positive malaria case was defined as one diagnosed by microscopy at the T&T-IVCD reference laboratory, employing the internationally recognized "gold standard" for malaria diagnosis [1].

"In-transit" referred to positive malaria cases among individuals who were ill on arrival and who spent any period of time at a T&T port of entry (for example, seafarers on cargo ships). Such a person received medical attention but did not remain within the country for the required one-year follow-up. During this time, it was possible for the *Anopheles* vectors present in the country to become infectious and then transmit malaria to the local population. An unknown origin of malaria is a case that usually occurs when the individual visited multiple malaria-endemic areas a few days apart prior to entering T&T.

The exclusion criteria were any person not diagnosed with malaria in T&T for the period 2009-2019 and illegal arrivals into T&T.

Data collection

T&T-IVCD

Data from T&T-IVCD were obtained from the Positive Malaria Cases Records Ledger, which records all positive malaria cases in the country. For each year, the following data variables were collected: sex (male and female), age group in years (1-20, 21-40, 41-60, 61-80, 81-100, and unknown), type of parasite infection (*P. vivax*, *P. falciparum*, *P. malariae*, and mixed), classification of case (imported, local, and unknown), and the country of origin of the infection.

Immigration Division

A census of the arrivals of nationals from Guyana and Venezuela to T&T was conducted for the period 2009-2019 by year and port of entry. This captured the volume and temporal distribution of these incoming nationals. Datasets were prepared for Guyana and Venezuela, respectively, with the headings "port of entry" and "year" (subheadings for each year from 2009 to 2019).

Data analyses

Data were analyzed using Microsoft Excel (Microsoft Corporation, Redmond, Washington) and IBM SPSS Statistics for Windows, Version 28 (Released 2021; IBM Corp., Armonk, New York). For manageability, "Country of origin of the infection" was organized into seven categories: Guyana, Venezuela, India, Sub-Saharan Africa, Trinidad, In-transit, and Unknown. The positive malaria cases from Sub-Saharan Africa were noted from Cameroon, Ghana, Guinea, Ivory Coast, Mali, Nigeria, Sierra Leone, Sudan, and Uganda.

Descriptive statistics were used to describe the parasite type and case classification of the positive malaria cases, which were presented in tabular form. The dataset comprised paired annual observations of confirmed positive malaria cases and corresponding international arrivals over the study period, which were plotted against each other to observe trends. The dataset comprised 11 paired observations from 2009 to 2019, with each pair representing the number of international arrivals and the corresponding number of confirmed positive malaria cases for that year. Data were complete with no missing values. Arrivals were disaggregated by nationality (Venezuela and Guyana) to allow for comparison with malaria cases. A dual-axis line graph was constructed to visually compare annual arrivals from Venezuela and Guyana (primary Y-axis) with the number of malaria cases (secondary Y-axis). The normality of the variables was assessed using skewness and kurtosis. A logarithmic transformation was applied to all variables to approximate normal distributions and reduce the influence of potential outliers.

Statistical analysis

Annual counts of malaria cases and international arrivals (Venezuela, Guyana) were log-transformed to improve normality and reduce leverage of outliers: X\* = log10(X). We assessed bivariate associations using Pearson product-moment correlation, reporting the corresponding statistic and degrees of freedom (df = n-2). Two-sided α = 0.05. For relationships reaching significance, we fit simple log-log linear regression models of the form $log10(Malaria Cases) = &beta;0+&beta;1 log10 (Arrivals)+&epsilon;$, reporting β1, R2, the model F statistic (1, df), and p-values. An exploratory model evaluated the combined log-transformed arrivals from Venezuela and Guyana.

## Results

Over the period 2009-2019, 211 positive malaria cases were diagnosed: 157 (74.4%) males and 54 (25.6%) females, with an age range of 3-81 years and a mean age of 35 years (SD=14.3). The age for one "in-transit" case was unknown, and their one-year follow-up was not completed.

Of the 211 positive malaria cases, the parasites identified were *P. vivax* 144 (68.2%), *P. falciparum* 47 (22.3%), *P. malariae* 12 (5.7%), and mixed infections 8 (3.8%) of both *P. vivax* and *P. falciparum* (Table 1). In 2009 and 2010, there were 24 and 23 diagnosed cases of malaria in T&T, respectively, and over the period 2011-2017, the highest was 19 in 2012 and the lowest was 8 in 2015. However, the number of diagnosed malaria cases increased to 40 in 2018 and 36 in 2019 (Table 1).

**Table 1:** Parasite type and case classification of the positive malaria cases in Trinidad and Tobago for the period 2009-2019 *Values are expressed as n (%). Categorical comparisons by chi-square (χ² = 32.39, df = 30, p = 0.35). Two-sided tests; significance threshold p < 0.05.

Year	Positive Cases	Parasite Type				Case Classification		
		*P. vivax*	*P. falciparum*	*P. malariae*	Mixed	Imported	Local	Unknown
2009	24	14 (58.3%)	4 (16.7%)	4 (16.7%)	2 (8.3%)	20 (83.3%)	4 (16.7%)	0 (0%)
2010	23	15 (65.2%)	6 (26.1%)	2 (8.7%)	0 (0%)	21 (91.3%)	2 (8.7%)	0 (0%)
2011	10	5 (50%)	4 (40%)	1 (10%)	0 (0%)	9 (90%)	1 (10%)	0 (0%)
2012	19	10 (52.6%)	7 (36.9%)	0 (0%)	2 (10.5%)	19 (100%)	0 (0%)	0 (0%)
2013	16	11 (68.8%)	4 (25%)	0 (0%)	1 (6.2%)	16 (100%)	0 (0%)	0 (0%)
2014	12	7 (58.4%)	3 (25%)	1 (8.3%)	1 (8.3%)	11 (91.7%)	1 (8.3%)	0 (0%)
2015	8	4 (50%)	4 (50%)	0 (0%)	0 (0%)	8 (100%)	0 (0%)	0 (0%)
2016	11	8 (72.7%)	2 (18.2%)	1 (9.1%)	0 (0%)	8 (72.7%)	1 (9.1%)	2 (18.2%)
2017	12	9 (75%)	3 (25%)	0 (0%)	0 (0%)	12 (100%)	0 (0%)	0 (0%)
2018	40	35 (87.5%)	3 (7.5%)	1 (2.5%)	1 (2.5%)	36 (90%)	2 (5%)	2 (5%)
2019	36	26 (72.2%)	7 (19.4%)	2 (5.6%)	1 (2.8%)	34 (94.4%)	2 (5.6%)	0 (0%)
Total	211	144 (68.2%)	47 (22.3%)	12 (5.7%)	8 (3.8%)	194 (91.9%)	13 (6.2%)	4 (1.9%)

There were 194 (91.9%) imported cases, 13 (6.2%) were acquired locally, and 4 (1.9%) were of unknown origin (Table 1). Of the 13 cases acquired locally, 11 were due to *P. malariae*, and the remaining two cases were due to *P. vivax*.

Of the 211 cases of malaria, these occurred among persons entering T&T from Guyana (77, 36.5%), Venezuela (75, 35.5%), Sub-Saharan Africa (30, 14.2%), India (9, 4.3%), in-transit (3, 1.4%), and unknown (5, 2.4%) (Table 2). Over the period 2009-2013, except in 2011, most of the positive malaria cases diagnosed in T&T had Guyanese country of origin. However, over the period 2016-2019, most of the positive malaria cases diagnosed in T&T had their country of origin in Venezuela, with 30 (75.0%) of 40 cases and 27 (75.0%) of 36 cases being diagnosed in 2018 and 2019, respectively (Table 2).

**Table 2:** Countries of origin of the positive malaria cases in Trinidad and Tobago for the period 2009-2019 Values are expressed as n (%). Country-of-origin distributions were compared across years using the chi-square (χ² = 253.37, df = 60, p < 0.001). Two-sided tests; significance threshold p < 0.05.

Year	Positive Cases	Guyana	Venezuela	India	Sub-Saharan Africa	In-Transit	Trinidad	Unknown
2009	24	17 (70.8%)	0 (0%)	2 (8.3%)	1 (4.2%)	0 (0%)	4 (16.7%)	0 (0%)
2010	23	17 (73.9%)	0 (0%)	2 (8.7%)	2 (8.7%)	0 (0%)	2 (8.7%)	0 (0%)
2011	10	2 (20%)	2 (20%)	3 (30%)	2 (20%)	0 (0%)	1 (10%)	0 (0%)
2012	19	12 (63.2%)	0 (0%)	1 (5.2%)	3 (15.8%)	3 (15.8%)	0 (0%)	0 (0%)
2013	16	14 (87.5%)	0 (0%)	0 (0%)	2 (12.5%)	0 (0%)	0 (0%)	0 (0%)
2014	12	5 (41.7%)	0 (0%)	0 (0%)	7 (58.3%)	0 (0%)	0 (0%)	0 (0%)
2015	8	0 (0%)	4 (50%)	0 (0%)	4 (50%)	0 (0%)	0 (0%)	0 (0%)
2016	11	2 (18.2%)	4 (36.3%)	0 (0%)	1 (9.1%)	0 (0%)	1 (9.1%)	3 (27.3%)
2017	12	1 (8.3%)	8 (66.7%)	1 (8.3%)	2 (16.7%)	0 (0%)	0 (0%)	0 (0%)
2018	40	5 (12.5%)	30 (75%)	0 (0%)	1 (2.5%)	0 (0%)	2 (5%)	2 (5%)
2019	36	2 (5.6%)	27 (75%)	0 (0%)	5 (13.8%)	0 (0%)	2 (5.6%)	0 (0%)
Total	211	77 (36.5%)	75 (35.5%)	9 (4.3%)	30 (14.2%)	3 (1.4%)	12 (5.7%)	5 (2.4%)

Table 3 shows there were 304,741 arrivals from Guyana for the period 2009-2019 at the different ports of entry. The majority (304,159, 99.8%) of these arrivals were in Trinidad, of which 288,789 (94.8%) arrived via airline flights to Trinidad, and there were 582 (0.2%) arrivals in Tobago. For the period 2009-2019, the number of arrivals from Guyana remained steady, with the highest at 33,447 in 2012 and the lowest at 23,241 in 2019 (Table 3).

**Table 3:** Arrivals from Guyana and Venezuela into Trinidad and Tobago for the period 2009-2019 Values are expressed as N (%) of total arrivals per year; summaries as mean ± SD. Differences between countries were analyzed via paired t-test (t(10) = 4.82, p = 0.0007). Two-sided tests; significance threshold p < 0.05.

Year	Guyana	Venezuela
2009	27,176 (67.1%)	13,349 (32.9%)
2010	25,322 (68.3%)	11,729 (31.7%)
2011	28,375 (70.1%)	12,130 (29.9%)
2012	33,447 (71.5%)	13,331 (28.5%)
2013	30,447 (68.6%)	13,941 (31.4%)
2014	27,281 (53.5%)	23,671 (46.5%)
2015	29,385 (47.2%)	32,913 (52.8%)
2016	28,889 (52.7%)	25,925 (47.3%)
2017	26,396 (60.9%)	16,982 (39.1%)
2018	24,882 (58.4%)	17,754 (41.6%)
2019	23,141 (76.9%)	6,962 (23.1%)
Total	304,741 (61.8%)	188,687 (38.2%)

There were 188,687 arrivals from Venezuela over the period 2009-2019 (Table 3) at the different ports of entry. The majority (188,378, 99.8%) of arrivals were in Trinidad, of which 158,428 (84.0%) arrived via airline flights in Trinidad, and there were 309 (0.2%) arrivals in Tobago. The number of arrivals per year from Venezuela remained steady, ranging from 11,729 to 13,942 during 2009-2013, then sharply increased, ranging from 23,671 to 32,913 during 2014-2016, and then steadily declined until 2019, when there were 6,962 arrivals (Table 3).

Over the period 2009-2019, there were 94.8% arrivals via airline flights from Guyana versus 84.0% from Venezuela, and the average number of arrivals from Guyana (mean = 27,703, SD = 2860) exceeded that from Venezuela (mean = 17,153, SD = 7524). The mean number of positive malaria cases was 19 (SD = 10.7) per year.

**Figure 1:**
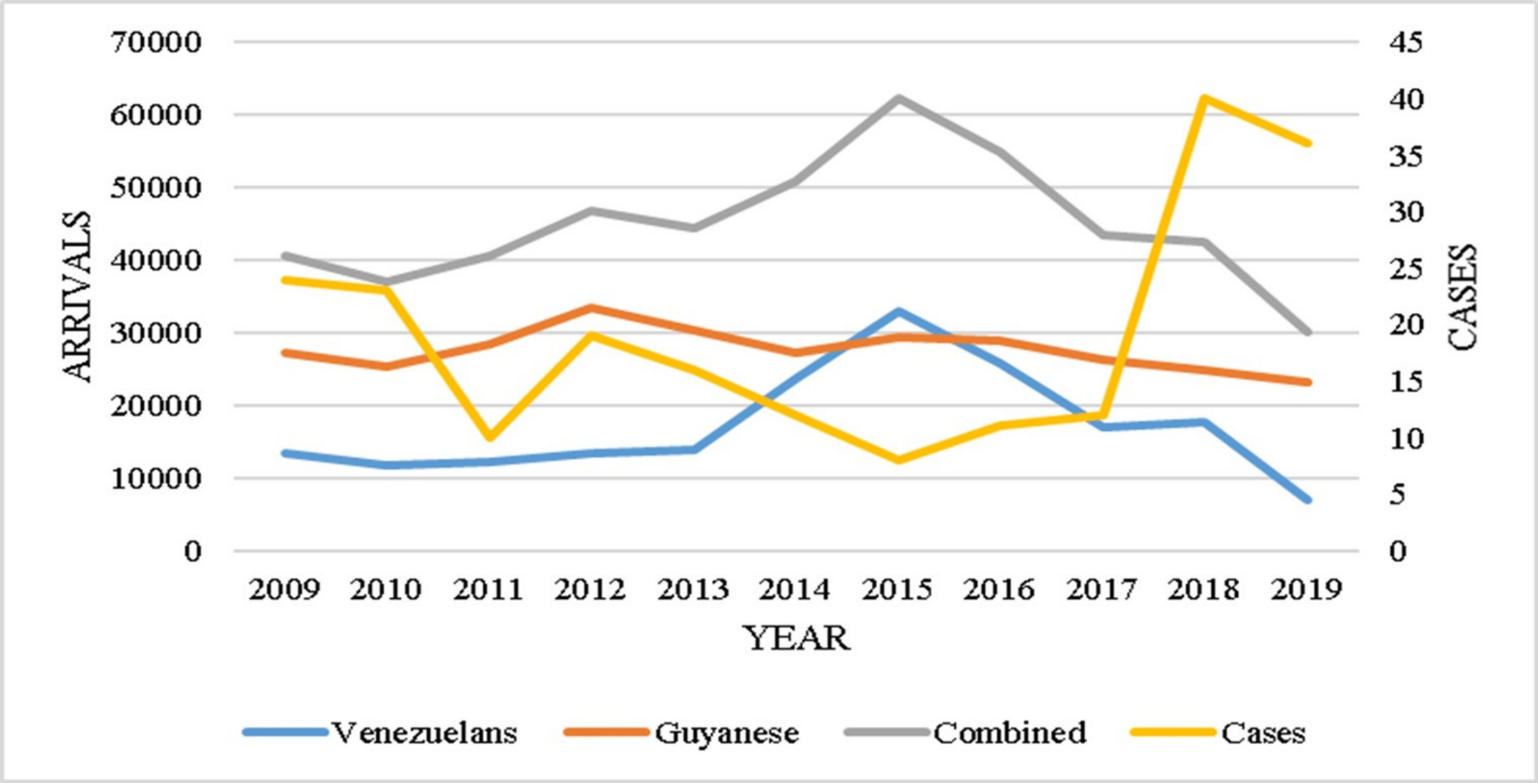
Line graph showing the linear relationship between the number of arrivals from Venezuela and Guyana, together with the number of positive malaria cases Lines depict annual arrivals (Venezuela, Guyana) on the left axis and malaria cases on the right axis; variables were log-transformed for correlation/regression analyses. Two-sided tests; significance threshold p<0.05.

Figure 1 presents a dual-axis line graph comparing annual arrivals from Venezuela and Guyana (primary Y-axis) with confirmed malaria cases (secondary Y-axis). The number of Guyanese arrivals appears to follow a similar trend to malaria incidence between 2010 and 2014. However, the number of malaria cases diagnosed in T&T sharply increased in 2018 and 2019.

Following log transformation, the distributions of Venezuelan arrivals, Guyanese arrivals, and positive malaria cases approximated normality. A Pearson product-moment correlation between log-transformed Guyanese arrivals and malaria cases yielded a moderate positive correlation (r = 0.552, t(9) = 1.99, p = 0.078; regression not pursued, R² = 0.305).

In contrast, the correlation between log-transformed Venezuelan arrivals and malaria cases was also moderate (r = 0.642, t(9) = 2.51, p = 0.033; simple regression significant with R² = 0.412 and F(1,9) = 6.31), indicating that Venezuelan arrivals significantly predict malaria incidence. The resulting regression model is Log-linear form: log(Malaria Cases) = -0.786 x log(Venezuelan Arrivals) + 4.526

Transformed back: $Malaria Cases = 92.388 x (Venezuelan Arrivals)-0.786$

When combining log-transformed arrivals from both Venezuela and Guyana, the correlation with malaria cases increased (r = 0.723, R² = 0.522, p = 0.052), indicating a strong positive relationship. However, the model did not reach statistical significance, and no further regression analysis was conducted.

## Discussion

The two highest countries of origin of patients diagnosed with imported malaria in the study were Guyana (36.5%) and Venezuela (35.5%), and the most prevalent parasite identified in the study was P. vivax (68.2%), the dominant malaria parasite present in Latin America, the Caribbean, and Asia [1]. The findings of the study have suggested that there is a statistically significant relationship (p = 0.033) between the number of positive malaria cases and the number of arrivals of persons from Venezuela in T&T for the period 2009-2019, indicating that Venezuelan arrivals significantly predict malaria incidence. Over the period 2016-2019, most positive malaria cases diagnosed in T&T had Venezuela as their country of origin, with 30 (75.0%) of 40 cases and 27 (75.0%) of 36 cases recorded in 2018 and 2019, respectively (Table 2). The number of arrivals from Venezuela (Table 3) almost doubled between 2014 and 2016, which coincided with the period of economic instability when many Venezuelan nationals left their country in search of better opportunities in other countries [18]. In Venezuela, over the period 2000-2019, the number of malaria cases increased approximately 13-fold, from 35,500 in 2000 to 467,000 in 2019 [9,19], with a rapid and significant increase since 2014 [9].

The unprecedented increase in malaria in Venezuela was attributed to the failure of the healthcare system, which resulted from the socio-economic collapse [20]. While the influx of Venezuelans entering T&T declined in 2017-2019, the government of T&T under the Migrant Registration Framework issued Minister's Permits to approximately 22,000 Venezuelan migrants to register to work within T&T in 2019 [21], and this number far exceeded the 6962 arrivals from Venezuela that year, suggesting the presence of undocumented Venezuelan migrants residing in T&T. Numerous media reports have also highlighted the frequent detention of boats with nationals of Venezuela by the Coast Guard when attempting to enter the country illegally, as well as those successfully landing undetected and persons then escaping to various parts of the country [8]. Therefore, the arrival data for Venezuelan nationals used in this study may be an underestimate.

A non-statistically significant (p = 0.078) relationship also existed between the number of positive cases of malaria and the number of arrivals from Guyana. It is possible that the figures for arrivals from Guyana are more accurate, as it is more challenging to enter T&T illegally from Guyana, whereas the journey from the Venezuelan coast is a mere 7 miles and can be easily traversed by simple vessels like pirogues. Guyana has made considerable progress in reducing the burden of malaria from a peak of approximately 60,000 cases in 1995 to a low of 13,200 cases in 2015 [22]. However, during the period 2015-2019, Guyana has experienced a greater than 40% increase in annual malaria cases, with over 20,000 cases reported in 2019 [1], most probably due to cross-border travel of migrants to participate in mining and logging in Guyana's remote interior border areas with its rainforest climate and high malaria burden coupled with difficulties in the delivery of services to these difficult-to-reach areas [22]. The healthcare system in Guyana provides malaria diagnosis, treatment, and care, as well as prevention services, to all persons residing in the country, including foreign nationals [22,23]. Since 2020, Guyana's economy has been transformed due to major offshore oil discoveries, becoming one of the fastest-growing economies in the world. Thus, due to oil revenues, the country is expected to increase its annual spending on malaria programs with the aim of elimination by 2030 [22].

Population movement is a key factor in malaria transmission [24-27], as the importation of malaria from endemic to non-endemic countries poses a risk of outbreaks. Of the 211 cases in our study, 194 (91.9%) were imported. Although T&T is certified "malaria-free," the presence of *Anopheles* mosquito vectors [6], diagnosed cases of malaria, and a susceptible local population provide the conditions necessary for successful malaria transmission to occur. Additionally, there was a greater than fourfold increase in malaria cases from 8 cases in 2015 to 36 cases in 2019 (Table 2); this sudden spike in imported cases is similar to that seen in Jamaica [28] and Greece [29], followed by local outbreak events in these malaria-free countries.

An undocumented status can deter individuals from seeking medical attention due to limited healthcare access, fear of arrest and deportation, and language barriers [30-32], with the consequence of late diagnosis of malaria. Some undocumented migrants have poor knowledge, attitudes, and practices towards malaria [30], resulting in individuals receiving incomplete courses of treatment due to poor adherence that may lead to relapse, resistance, and onward transmission of malaria [30]. Due to fear of deportation, there have been reports that patients abscond from care and treatment; some frequently move residences [33] and regularly change their phone numbers, making follow-up difficult. This issue presents a crucial problem, as *P. vivax* is the most challenging malaria parasite to treat. With improper treatment and follow-up, there may be an increased risk of relapse and local transmission [34]. It is recommended that the use of rapid diagnostic tests for malaria should be implemented at the point of entry into T&T, as this allows a quick and accurate diagnosis and may be used to screen migrants and travelers, thereby enabling prompt treatment and reducing the introduction and transmission of imported malaria [30]. For those migrants who experience barriers to accessing healthcare, rapid diagnostic testing for malaria is especially useful when integrated into community-based programs by engaging non-governmental organizations and local community leaders [30]. Such collaborations can facilitate malaria testing for new migrants (including some with undocumented status) and enhance reporting of suspected malaria cases [30].

It is important that T&T develops a web-based data management platform to replace the current paper-based system to improve malaria reporting and management [35]. This is consistent with the WHO guidelines for malaria elimination, which advocate improved malaria surveillance and the implementation of a rapid, accurate reporting system to reduce local transmission, employ targeted vector control interventions, and conduct contact transmission assessments [1,35].

Strengthening intersectoral cooperation is important for malaria control, as mobile migrant populations represent a substantial reservoir for transmission. This involves the engagement of various stakeholders and sectors beyond the Ministry of Health, including the Ministries of Agriculture, Education, and the Environment, and community-based organizations to implement comprehensive and sustainable strategies to address the complex issues that impact malaria transmission [36]. A study conducted by Ng'ang'a et al. in Kenya [37] demonstrated that intersectoral collaboration via advocacy and social mobilization improved the integrated vector management strategy through enhanced capacity building, community partnership, optimum use of resources, and participation in malaria control [37]. A systematic review conducted by Naing et al. also showed that an intersectoral approach among mobile migrant populations in Myanmar supported the use of insecticide-treated nets for malaria prevention [38].

There are several limitations of the study; the number of Venezuelan arrivals in T&T and the number of malaria cases reported over the study period may be an underestimate, as many individuals from Venezuela enter T&T illegally, and some undocumented migrants who may be infected with malaria may not seek healthcare due to fear of arrest and deportation. The study used secondary data that were not collected to answer the research question; thus, our researchers had no control over the study population or the variables of interest, including sociodemographic information, which was lacking in the study. Secondary data analysis is observational and retrospective and not the appropriate type of study to determine causal relationships. As an ecological analysis using aggregated data, it cannot infer individual-level causality and may be subject to ecological fallacy. Potential confounders such as vector control measures, seasonal trends, and diagnostic changes were not controlled for. Future research incorporating more granular temporal data, individual-level exposure information, and multivariable modelling could yield more robust and interpretable findings, as the COVID-19 pandemic may affect malaria control programs.

## Conclusions

Population movement from high malaria risk areas may increase the likelihood of transmission in T&T, and in our study, the influx of individuals from Venezuela significantly predicted malaria incidence. Rapid diagnostic testing and treatment of symptomatic individuals from endemic countries, along with malaria literacy, health education, surveillance, and vector control, are some of the interventions urgently needed to prevent a malaria resurgence.
